# Phylogenetic diversity of functional genes in deep-sea cold seeps: a novel perspective on metagenomics

**DOI:** 10.1186/s40168-023-01723-7

**Published:** 2023-12-15

**Authors:** Danrui Wang, Jiangtao Li, Lei Su, Wenli Shen, Kai Feng, Xi Peng, Zhujun Wang, Bo Zhao, Zheng Zhang, Zhaojing Zhang, Étienne Yergeau, Ye Deng

**Affiliations:** 1 CAS Key Laboratory of Environmental Biotechnology, Research Center for Eco-Environmental Sciences, Chinese Academy of Sciences, Beijing, 100085 China; 2 College of Resources and Environment, University of Chinese Academy of Sciences, Beijing, 100049 China; 3 State Key Laboratory of Marine Geology, Tongji University, Shanghai, 200092 China; 4 Institute of Marine Science and Technology, Shandong University, Qingdao, 266237 China; 5 College of Tropical Crops, Hainan University, Haikou, 572000 China; 6 Institut National de La Recherche Scientique, Centre Armand-Frappier Santé Biotechnologie, Laval, H7V 1B7 QC China

**Keywords:** Cold seep, Phylogenetic diversity, Metagenomics, Functional genes

## Abstract

**Background:**

Leakages of cold, methane-rich fluids from subsurface reservoirs to the sea floor are termed cold seeps. Recent exploration of the deep sea has shed new light on the microbial communities in cold seeps. However, conventional metagenomic methods largely rely on reference databases and neglect the phylogeny of functional genes.

**Results:**

In this study, we developed the REMIRGE program to retrieve the full-length functional genes from shotgun metagenomic reads and fully explored the phylogenetic diversity in cold seep sediments. The abundance and diversity of functional genes involved in the methane, sulfur, and nitrogen cycles differed in the non-seep site and five cold seep sites. In one Haima cold seep site, the divergence of functional groups was observed at the centimeter scale of sediment depths, with the surface layer potentially acting as a reservoir of microbial species and functions. Additionally, positive correlations were found between specific gene sequence clusters of relevant genes, indicating coupling occurred within specific functional groups.

**Conclusion:**

REMIRGE revealed divergent phylogenetic diversity of functional groups and functional pathway preferences in a deep-sea cold seep at finer scales, which could not be detected by conventional methods. Our work highlights that phylogenetic information is conducive to more comprehensive functional profiles, and REMIRGE has the potential to uncover more new insights from shotgun metagenomic data.

Video Abstract

**Supplementary Information:**

The online version contains supplementary material available at 10.1186/s40168-023-01723-7.

## Background

Within the last few decades, targeted amplicon sequencing and shotgun sequencing have been used to detect the composition and potential functions of microbial communities. 16S rRNA gene sequencing is the most widely used amplicon sequencing method. Although several tools, such as PICRUST2 and Tax4Fun2, make it possible to predict functional profiles from 16S rRNA gene sequencing data, these predictions tend to be biased towards known reference genomes, and cannot provide the resolution to perform strain-based analyses [1, 2]. Moreover, some relatively conserved functional genes can also be used as marker genes for certain functional groups. Nevertheless, low primer coverage is a common issue [3]. In comparison, shotgun metagenomic methods provide unbiased detection of various microbial groups and help to understand microbial diversity and functional profiles. Even so, many metagenomic tools rely heavily on reference databases, and often cannot represent the true phylogeny of the studied ecosystems [4, 5].

Fully exploiting the totality of information provided by metagenomes, rather than focusing solely on deposited sequences of known genes, is essential for revealing the taxonomic and functional compositions of microbial communities in unexplored natural environments. To obtain the real species composition from shotgun reads, researchers have developed EMIRGE (expectation maximization iterative reconstruction of genes from the environment), which enabled the reconstruction of full-length ribosomal genes from microbial communities [6]. However, EMIRGE was designed for small subunit ribosomal RNA (SSU rRNA) genes, with SILVA being the only compatible database. Therefore, broadening the applicability of this tool for comprehensive analysis of diverse functional genes necessitates further refinement and improvement.

Marine sediments are typical underexplored natural environments. Normally, in continental slopes, methane from the subsurface diffuses upwards slowly and is oxidized by microbes before reaching the sediment–water interface [7]. However, despite the more abundant and richer microbiota, only 20–80% of the methane can be consumed in deep-sea cold seep areas due to the high methane flux and fluid flow rate [8]. The biological consumption of methane in the ocean is comprised of aerobic and anaerobic oxidation [9, 10]. Aerobic oxidation of methane (AeOM) is limited to the top oxygenated sediments [11], while anaerobic oxidation of methane (AOM), mainly driven by consortia of anaerobic methanotrophic archaea (ANME) and sulfate-reducing bacteria (SRB), dominates methane consumption in marine ecosystems, accounting for 75–90% of methane consumption [12, 13]. Previous studies indicated that the AOM process could also couple to denitrification [14, 15], or provide ATP for nitrogen fixation [16]. Consequently, the methane cycle of cold seeps is inextricably linked to the sulfur and nitrogen cycles.

Owing to the reduced biological complexity of microbial communities and tight coupling between geochemical and biological processes, cold seeps are ideal targets for the study of composition, function, evolution, and environmental adaptation [17]. In 2015, the Haima remote-operated vehicle discovered a large active cold seep site (known as Haima cold seep) on the northern continental slope of the South China Sea. Subsequent dives showed that the site consisted of at least six individual patches of communities and displayed noticeable ecological and geochemical gradients [13, 18]. However, our understanding of the phylogeny of functional genes and the interactions between microbial functional groups in these systems remains poor.

In this study, we sampled a cold seep site (CS) and an adjacent non-seep background site (NS) by a push-core sampler in the Haima cold seep area. To retrieve full-length functional genes from raw metagenomic reads, we developed REMIRGE (Reprogrammed EMIRGE), and then used it to analyze representative functional genes of the methane, sulfur, and nitrogen cycles, constructed maximum-likelihood trees, calculated the indices of phylogenetic diversity, and analyzed the correlation between representative functional genes. Metagenomic datasets from four other cold seeps in the South China Sea were also included for analysis and comparison. In summary, we used REMIRGE to delve into the metabolic pathways and diversity profiles of functional genes and retrieved new insights into the cold-seep microbial communities. Furthermore, this method holds significant promise for investigating microbial functional groups in a variety of diverse habitats.

## Results

### REMIRGE and the online platform

**Fig. 1:**
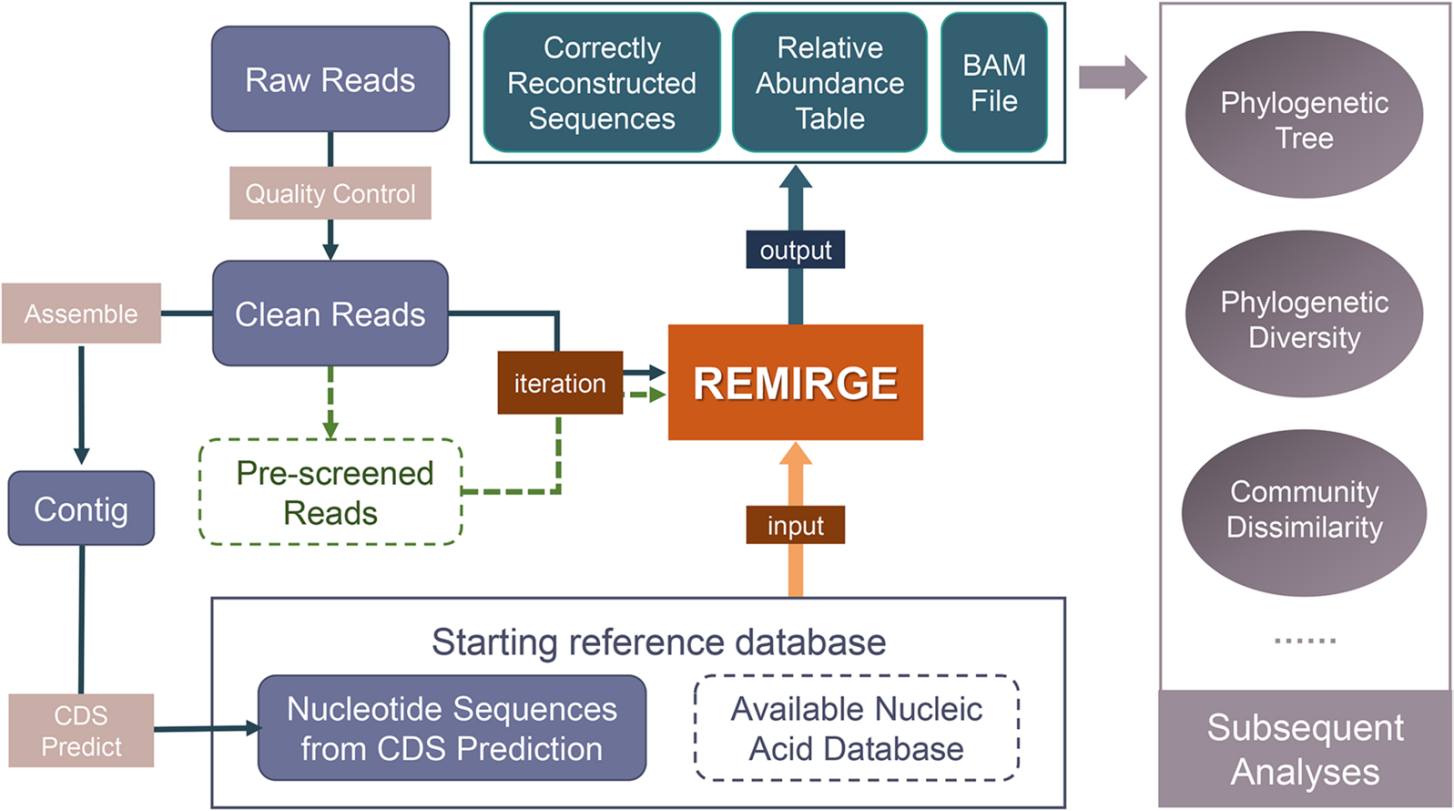
Flowchart for REMIRGE. REMIRGE requires a FASTA-formatted starting reference database and FASTQ-formatted clean read files. The starting reference database includes nucleotide sequences from CDS prediction or available nucleic acid databases. The optional pre-screening step can significantly reduce the running time. After several iterations, the correctly reconstructed sequences, relative abundance table, and BAM-formatted file are output for various subsequent analyses

Our REMIRGE program was built upon the core algorithm of EMIRGE, which employs an iterative method based on the expectation–maximization algorithm [6]. The analysis pipeline is illustrated in Fig. 1. REMIRGE requires FASTQ-formatted read files and a FASTA-formatted reference database as inputs. Clean reads after quality control can be directly input for REMIRGE, or alternatively, a pre-screening step can be implemented to identify potential target reads, expediting program execution. The initial reference database can be chosen from the predicted coding sequences (CDS), an existing reference database such as FunGene [19], or a combination of both. The outputs include correctly reconstructed sequences in FASTA format, the overall relative abundance of these sequences, and a BAM-formatted file generated by the last iteration. Furthermore, abundance calculation and normalization scripts can transform the outputs into results supporting various subsequent analysis methods. All necessary scripts are provided on GitHub to facilitate localized analysis. Additionally, our visual online analysis platform (https://remirge.denglab.org.cn) offers a convenient solution for users lacking analysis resources.

## Characteristics and microbial communities of the Haima cold seep

**Fig. 2:**
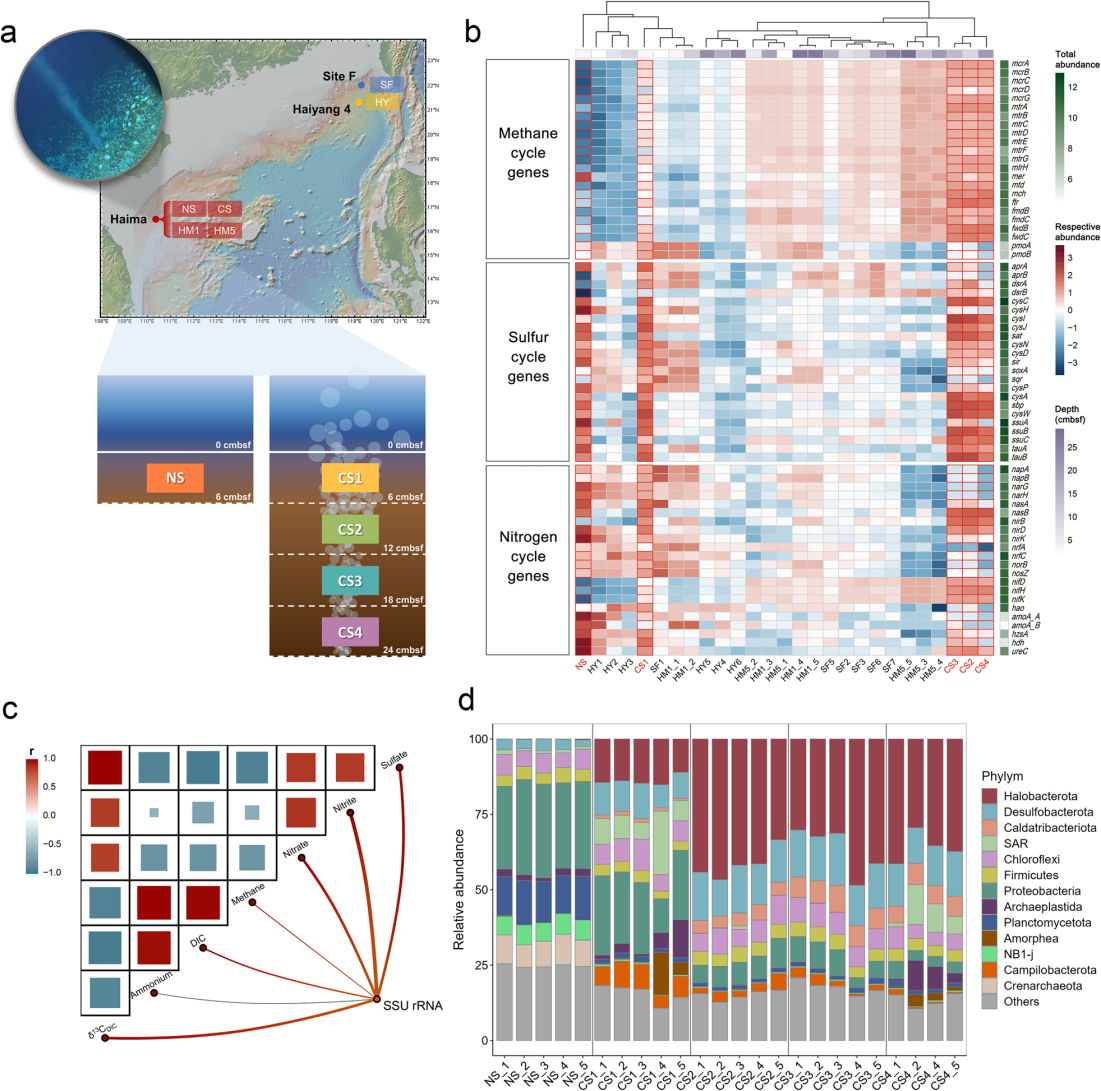
Schematic map of sampling sites and profiles of microbial communities. **a** The locations of Haima, Site F, and Haiyang 4 cold seeps, constructed with GeoMapApp (www.geomapapp.org). In our sampled cold seep site (CS), gas bubbles were found at the center of a dense mussel bed. At the CS site, sediment was evenly divided into four layers, meanwhile the non-seep (NS) site was collected from the adjacent non-cold seep background. Each layer had five replicates. **b** The abundance of genes implicated in methane, sulfur, and nitrogen cycles at the non-seep and five cold seep sites. Each row represents a gene and each column represents a sampling layer. For each gene, the total abundance of all samples was logarithmically transformed and shown on the right side (white-green block). The respective abundances of samples were logarithmically transformed and scaled by row (blue-red block). The depths of samples are shown on the top (white-purple block). **c** Mantel test between environmental factors and SSU rRNA gene. Red curves indicate significant correlations (*p* < 0.05), while gray curves indicate insignificant correlations (*p* > 0.05), and the line width represents the *r* statistic of the Mantel test. Pairwise comparisons of environmental factors were conducted and shown in the heat map. The color gradients and size of the squares represent Pearson correlation coefficients. **d** The stacked bar chart at the phylum level, based on annotations and respective abundance of SSU rRNA gene sequence clusters

In 2019, we collected sediment cores from the Haima cold seep and an adjacent non-seep site. The 24 cm sediment core from the methane-rich cold seep was equally divided into four layers (CS1-4), while the 6 cm surface layer of the non-seep site (NS) was used as the control (Fig. 2a). Each layer had five replicates. Both NovaSeq and Nanopore technologies were applied for shotgun sequencing, generating reads for hybrid metagenomics assembly. Simultaneously, another 24 cm sediment core was divided into 12 layers to measure environmental factors. According to the Mantel analysis, most environmental factors were significantly correlated with the SSU rRNA gene, indicating that multiple environmental factors collectively influence the microbial communities in the Haima cold seep (Fig. 2c).

As sediment depth increased, we observed an exponential increase in dissolved inorganic carbon (DIC) (Figure S1f). Generally, the source of DIC in deep-sea sediments could be attributed to either anaerobic oxidation of methane or organoclastic sulfate reduction (OSR) [20]. Both processes utilize sulfate as an electron acceptor, convert carbon in methane or other complex organic compounds into DIC, and consequently contribute to the permanent carbon sink in the deep sea [21]. In contrast to other organic sources, methane has significantly lower δ^13^C content. The extremely low δ^13^C_DIC_ in pore water indicates that AOM is the primary carbon source of DIC (Figure S1g) [22]. Moreover, the methane concentration increased exponentially while sulfate concentration decreased linearly with depth (Figure S1a and b). This pattern is a typical characteristic of the sulfate-methane transition zone (SMTZ), and the shallow SMTZ in this site implied a large methane flux, accompanied by active sulfate-dependent anaerobic methane oxidation (SAMO). Although nitrate and nitrite also exhibited decreasing trends with depth, their concentrations are relatively low (less than 5 μM) (Figure S1c and d), suggesting that denitrification-coupled AOM may be limited.

In addition, annotations at the phylum level revealed that Proteobacteria is the most abundant phylum in the surface layers (NS and CS1) and is widely distributed across all samples (Fig. 2d). Halobacterota (includes ANME) and Desulfobacterota (includes SRB) were the two most abundant phyla in the cold seep, especially in the subsurface layers. In summary, environmental data and taxonomic information confirmed that sulfate-dependent anaerobic methane oxidation, mediated by ANME and SRB, is the dominant biogeochemical process at the Haima cold seep site.

## Geographically different functional genes in cold seeps

Besides CS, datasets of four other cold seep sites in the South China Sea (HY, SF, HM1, and HM5, see Data S1 for details) were also included for primary analysis and comparison of representative functional genes. Next, we used MCycDB [23], SCycDB [24], and NCycDB [25] to calculate the abundances of individual samples (blue-red blocks), as well as the total abundance (white-green blocks) of each functional gene (Fig. 2b). Most of the genes related to AOM, including *mcrABCDG*, *mtrABCDEFGH*, *mer*, *mtd*, *mch*, *ftr*, and *fmdBC* (or *fwdBC*), were abundant in the cold seeps, peculiarly in the deeper layers. *dsrAB*, which are involved in dissimilatory sulfate reduction, were also abundant in the cold seeps. Most representative genes for the nitrogen cycle were relatively diverse and abundant in the surface and shallow layers, especially *napAB*, *narGH*, *norB*, and *nosZ* for denitrification. Notably, *nifDKH* genes for nitrogen fixation were more abundant in the deep layers of the cold seeps. To better measure the diversity profiles of functional genes in the five cold seep sites, we proposed a framework in units of gene sequence clusters (GSCs) using REMIRGE. REMIRGE generated the sequences of reconstructed GSCs and respective abundance, making it possible to build phylogenetic trees and calculate the weighted Unifrac β-diversity among samples. We also adopted the use of Hill numbers, for both taxonomic diversity (^*q*^*D*) and phylogenetic diversity (^*q*^*PD*), to quantify the diversity of functional genes based on GSCs [26]. The diversity order *q* determines the sensitivity to relative abundances [27].

**Fig. 3:**
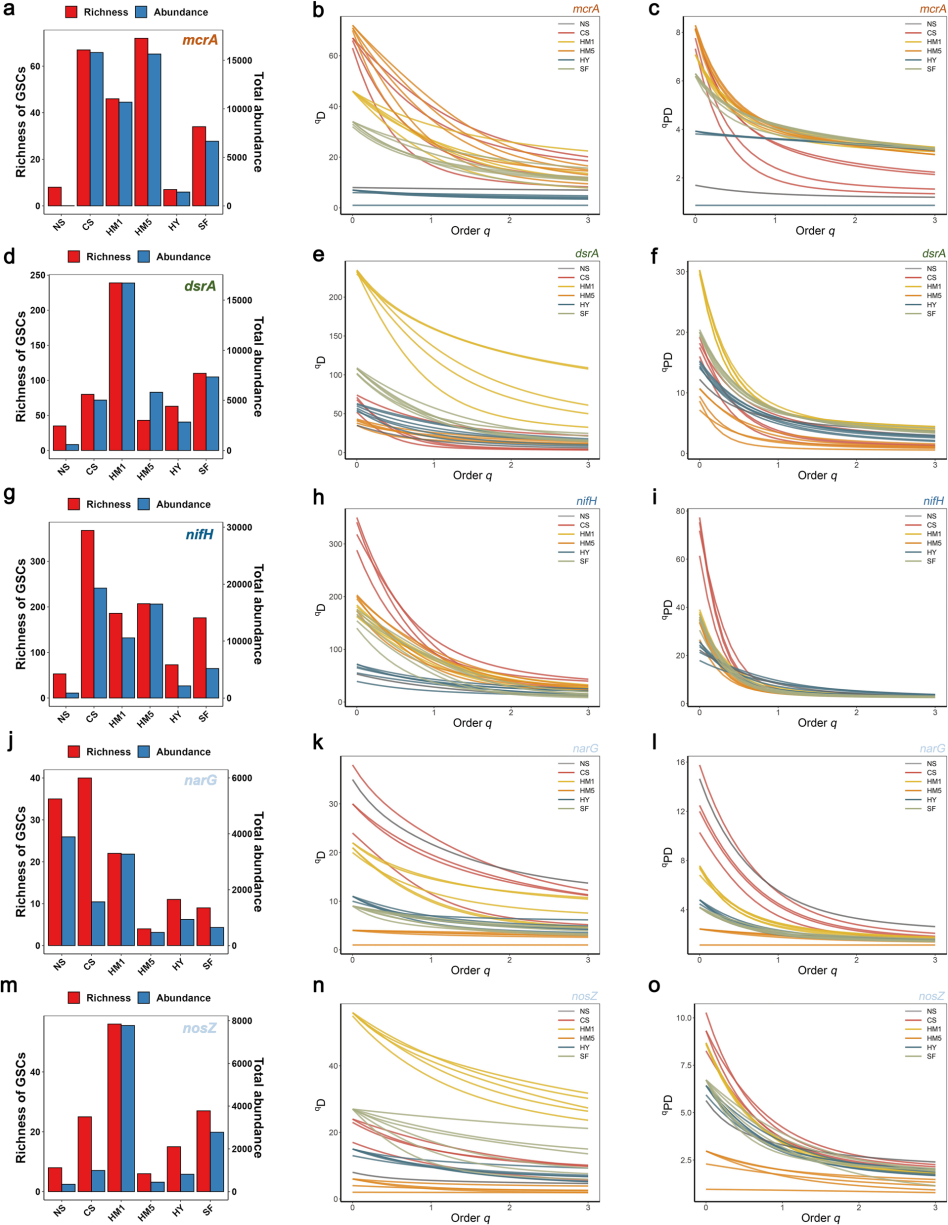
Richness, abundance, and diversity profiles of representative genes in different cold seep sites**.** In the bar graphs of functional genes, the red bars on the left signify richness, which is scaled with the left *y*-axis, representing how many distinct GSCs are present for that particular functional gene. The blue bars on the right signify abundance, which is scaled with the right y-axis, indicating the average number of reads that successfully mapped to the reference sequences of the corresponding functional gene at the cold seep site. The middle ^*q*^*D* profiles depict the taxonomic diversity, specifically referring to the diversity of GSCs, while the ^*q*^*PD* profiles on the right represent the phylogenetic diversity. Different colors of the curves correspond to distinct cold seep sites. ^*q*^*D* and.^*q*^*PD* vary in order q which determines the measures’ sensitivity to GSC relative abundances. The weight assigned to GSCs with higher relative abundance augments as the q-value increases. Smooth curves indicate high evenness. The colors of functional gene names represent the metabolic processes in which they participate. **a**–**c ***mcrA* for anaerobic oxidation of methane (orange). **d**–**f ***dsrA* for dissimilatory sulfate reduction (green). **g**–**i ***nifH* for nitrogen fixation (blue). **j**–**l ***narG*, and **m**–**o ***nosZ* for denitrification (light blue)

The PCoA plots of five representative functional genes, including *mcrA*, *dsrA, nifH*, *narG*, and *nosZ*, showed that different cold seep sites were separated (Figure S2, *P* < 0.001, PERMANOVA). Although the richness and abundance of the *mcrA* gene exhibited substantial variation across the cold seep sites, the ^*q*^*PD* profiles of the three sites from the Haima cold seep (CS, HM1, and HM5) were relatively similar (Fig. 3a–c), among which CS showed a steeper slope, indicating that abundance of the *mcrA* GSC was more uneven. Additionally, CS and HM5 showed higher diversity and abundance of *mcrA*, *dsrA*, and *nifH*, indicating the presence of AOM, sulfate reduction, and nitrogen fixation processes (Fig. 3a–i). In contrast, the low abundance and diversity of *narG* and *nosZ* observed at HM5 suggested an absence of denitrification processes (Fig. 3j–o). At the HM1 site, the high abundance and diversity of *dsrA*, *narG*, and *nosZ* demonstrated the significance of sulfate reduction and denitrification processes (Fig. 3).

## Divergent functional genes in different sediment depth

**Fig. 4:**
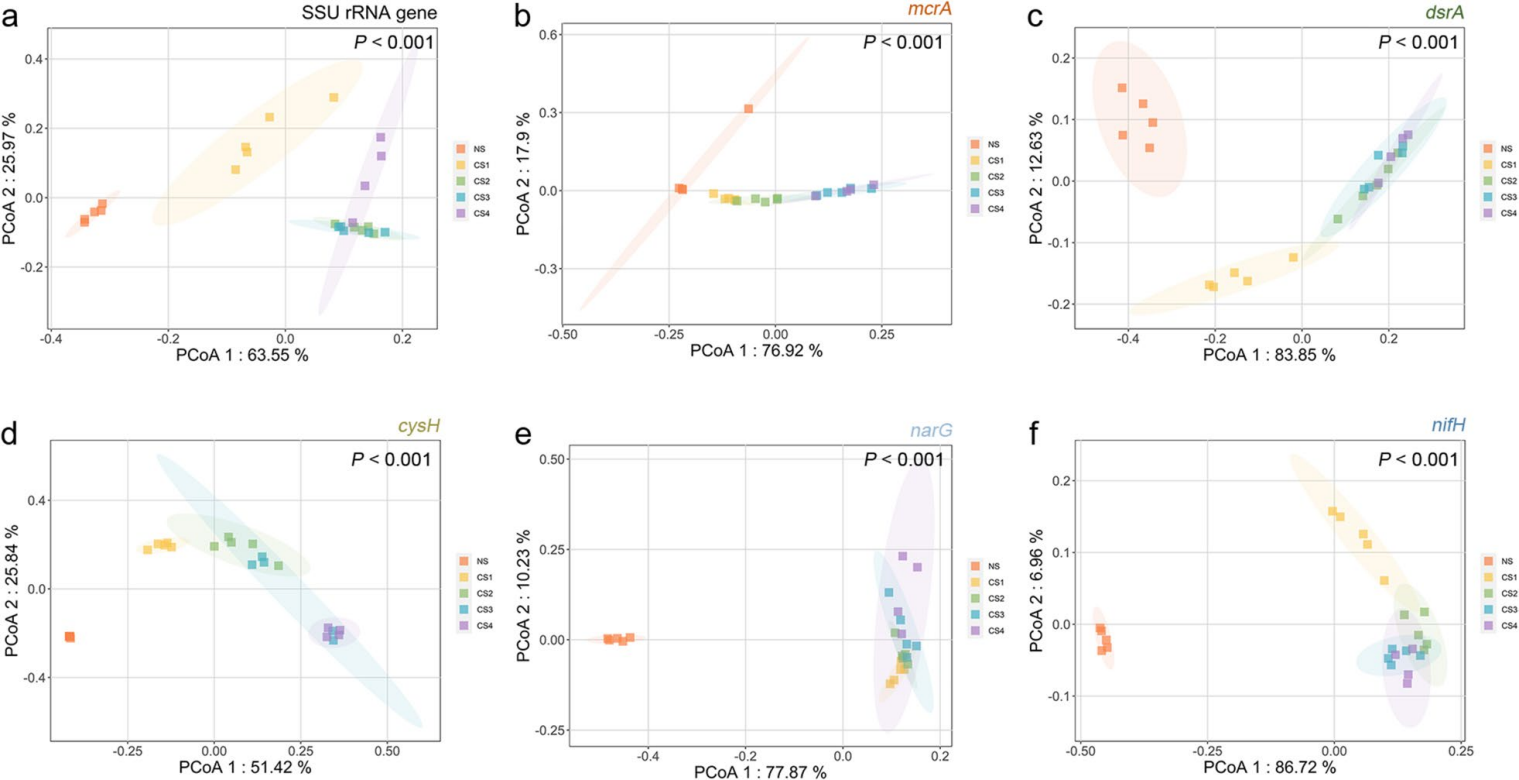
Principal coordinates analysis (PCoA) plots of representative genes based on weighted-Unifrac distance. Ellipses represent 95% confidence intervals. The colors of functional gene names represent the metabolic processes in which they participate. **a** SSU rRNA gene. **b ***mcrA* for anaerobic oxidation of methane (orange). **c ***dsrA* for dissimilatory sulfate reduction (green). **d ***cysH* for assimilatory sulfate reduction (yellow-green). **e ***narG* for denitrification (light blue). **f ***nifH* for nitrogen fixation (blue). Overall *P* values are annotated on the plots, while pairwise *P*- and F- values are compiled in Data S3

Sampling site CS exhibited diverse genes involved in the methane, sulfur, and nitrogen cycles (Fig. 2b), suggesting that CS harbored a wide range of functional groups with great metabolic potential. Notably, there were few significant differences in the abundances of representative functional genes among sediment layers (Data S2, Dunn's all-pairs rank comparison test), but the newly developed REMIRGE revealed more obvious phylogenetic divergence. For most AOM-related functional genes (Fig. 4 and Figure S3), NS, CS1, CS2, and CS3-4 formed four distinct clusters. This pattern was also observed in the *nifH* PCoA plot. Meanwhile, *sat*, *aprA*, and *dsrA*, representative functional genes of three main steps in dissimilatory sulfate reduction, showed three distinct clusters formed by NS, CS1, and layers CS2-4. This pattern was also observed in the *narG* and *nosZ* genes, illustrating that functional groups of dissimilatory sulfate reduction and denitrification were significantly different in the surface and subsurface layers.

**Fig. 5:**
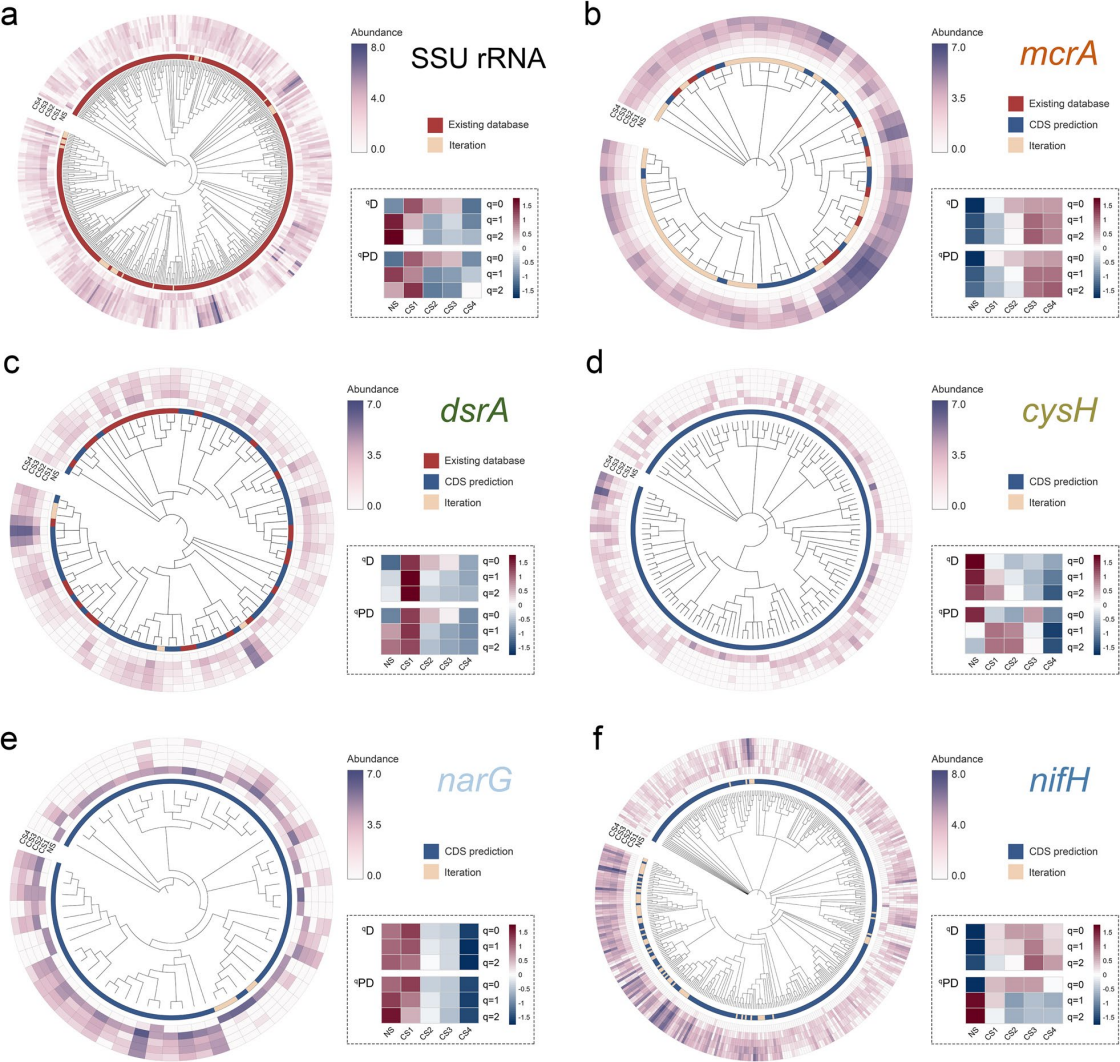
Phylogenetic trees, abundance heat maps, and diversity profiles of representative genes in different layers of the CS site. Only branches whose supporting values were higher than 0.75 are represented in the phylogenetic trees. The outer heat maps illustrate logarithmically transformed abundance. The inner strips represent the types of gene sequence clusters (GSCs), while the heat maps at the bottom-right inset for each gene show the diversity profiles measured by Hill numbers, logarithmically transformed, and scaled by row. The colors of functional gene names represent the metabolic processes in which they participate. **a** SSU rRNA gene. **b ***mcrA* for anaerobic oxidation of methane (orange). **c ***dsrA* for dissimilatory sulfate reduction (green). **d ***cysH* for assimilatory sulfate reduction (yellow-green). **e ***narG* for denitrification (light blue). **f ***nifH* for nitrogen fixation (blue)

The phylogenetic diversity of GSC was further demonstrated (Fig. 5). We found that most SSU rRNA GSCs (92.1%) were from the SILVA 138 database (red coloration) (Fig. 5a), while 7.9% could be still generated by iteration (yellow coloration). Compared with the SSU rRNA gene (7.9%, Fig. 5a) and *dsrA* (4.4%, Fig. 5c), *mcrA* GSCs generated from iteration accounted for a far larger proportion (56.7%, Fig. 5b). Most likely because the existing *mcrA* database was incomplete, the iteration method discovered many new *mcrA* GSCs. *mcrA* gene appeared to be more diverse in the cold seep, especially the subsurface layers (Fig. 5b). However, *dsrA* was more diverse in the surface layer (Fig. 5c). *cysH* and *sir* genes were more diverse in NS and CS1, implying that the surface layers of the cold seep and the non-seep site had greater functional potentials for assimilatory sulfate reduction (Fig. 5d and Figure S4i). As for denitrification, *narG*, responsible for nitrate reduction to nitrite, was diverse in NS and CS1, while *nosZ*, responsible for N_2_O reduction to N_2_, was diverse in CS1 (Fig. 5e and Figure S4k). Also, *nifH*, the marker gene for nitrogen fixation, was diverse in the cold seep (Fig. 5f). In general, functional genes related to AOM and nitrogen fixation were more diverse in the cold seep’s subsurface layers, while most other genes were more diverse in the surface layer.

## Coupling between specialized gene sequence clusters

Mantel analysis revealed that the *mcrA* gene strongly correlated with the sulfate concentration, while the *dsrA* gene seemed unrelated to the methane concentration (Table S1). This observation suggests that AOM largely relies on sulfate concentrations, while sulfate reduction is relatively independent. Thus, we hypothesize that SAMO is the primary methane consumption process, with only a few SRB participants.

**Fig. 6:**
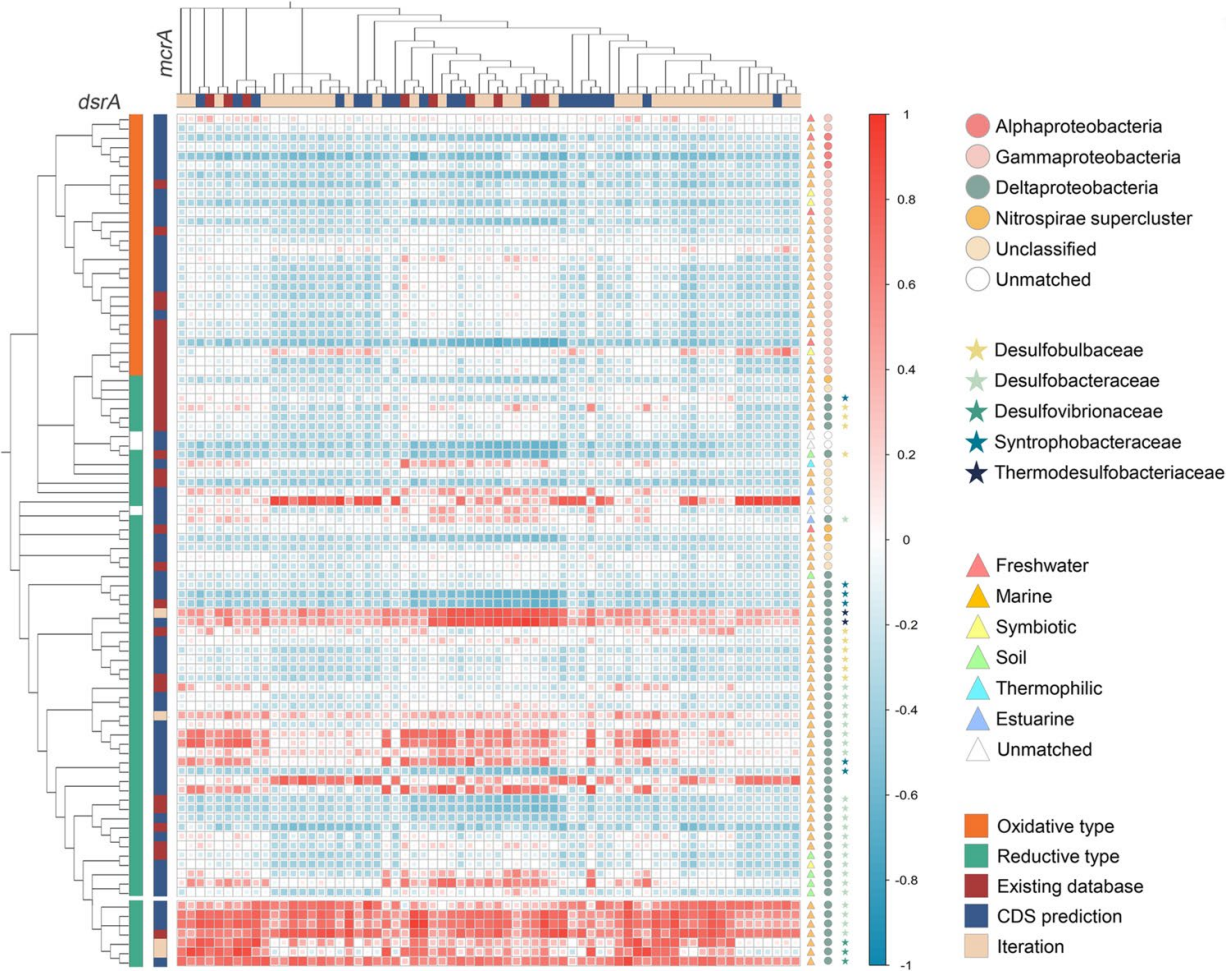
Pearson correlation analysis between *dsrA* and *mcrA*. Strips on the top and left represent the types of gene sequence clusters (GSCs). Annotation results of *dsrA* are shown on the right side. Triangles represent distributions, circles represent the annotations at the class level, and stars represent the annotations of Deltaproteobacteria at the family level

Correlation analyses have provided information about co-occurrences between operational taxonomic units (OTUs) or functional groups, which is an essential precondition of coupling or symbiosis [28]. To test the aforementioned hypothesis and gain deeper insights into the microbial consortia of ANME and SRB in the CS site, we analyzed the correlation between *mcrA* and *dsrA* genes, and used a well-organized *dsrAB* database to annotate the type, distribution, and taxon of each *dsrA* GSC [29]. About 77% of the *dsrA* GSCs were derived from marine environments while 23% of lineages were originally recovered from other environments (Fig. 6), suggesting both marine-specific *dsrA* GSCs and widely distributed *dsrA* GSCs were present in our sampling site. The majority of oxidative-type *dsrA* GSCs were negatively correlated to *mcrA*, while reductive-type *dsrA* GSCs were negatively or positively correlated (Fig. 6). Markedly, a distinct clade positioned at the bottom of Fig. 6 encompassed six *dsrA* GSCs that exhibited strong correlations with the majority of *mcrA* GSCs, indicating a shared ecological niche between SRB possessing specific dsrA GSCs and ANME. This special *dsrA* clade was annotated as either Desulfobacteraceae (known ANME partner) or Desulfovibronaceae (an electroactive microorganism) [30, 31]. However, not all *dsrA* GSCs annotated as Desulfobacteraceae or Desulfovibronaceae were clustered together, with some GSCs displaying negative correlations with *mcrA* GSCs. We inferred that not all complementary functional groups coupled, with specific members of the SRB possessing the potential to couple with a broad spectrum of ANME, allowing them to dominate the SAMO process.

By contrast, there were clear negative correlations between almost all *mcrA* and *narG* GSCs (Figure S5), suggesting that nitrate reduction was unlikely to be coupled with AOM. Additionally, a clade of *nifH* (located on the right side of Figure S6b) had strong positive correlations with most *mcrA* GSCs, as well as with the small clade of *dsrA* (bottom right of Figure S6a). Previous studies have shown that while biological nitrogen fixation can be fueled by various catabolic processes, nitrogen fixation pathways correlated with AOM were selected for in hydrocarbon seeps [16]. Our results further indicated the unique diversity of diazotrophs in the CS site and uncovered the dominant GSCs. The *nifH* clade on the right side of Figure S6b, which was strongly associated with *mcrA* genes, might contain the most dominant GSCs in this symbiotic system, while the remaining *nifH* GSCs indicated nitrogen fixation pathways driven by other catabolic processes.

## Functional pathway preferences in the cold seep and the non-seep site

**Fig. 7:**
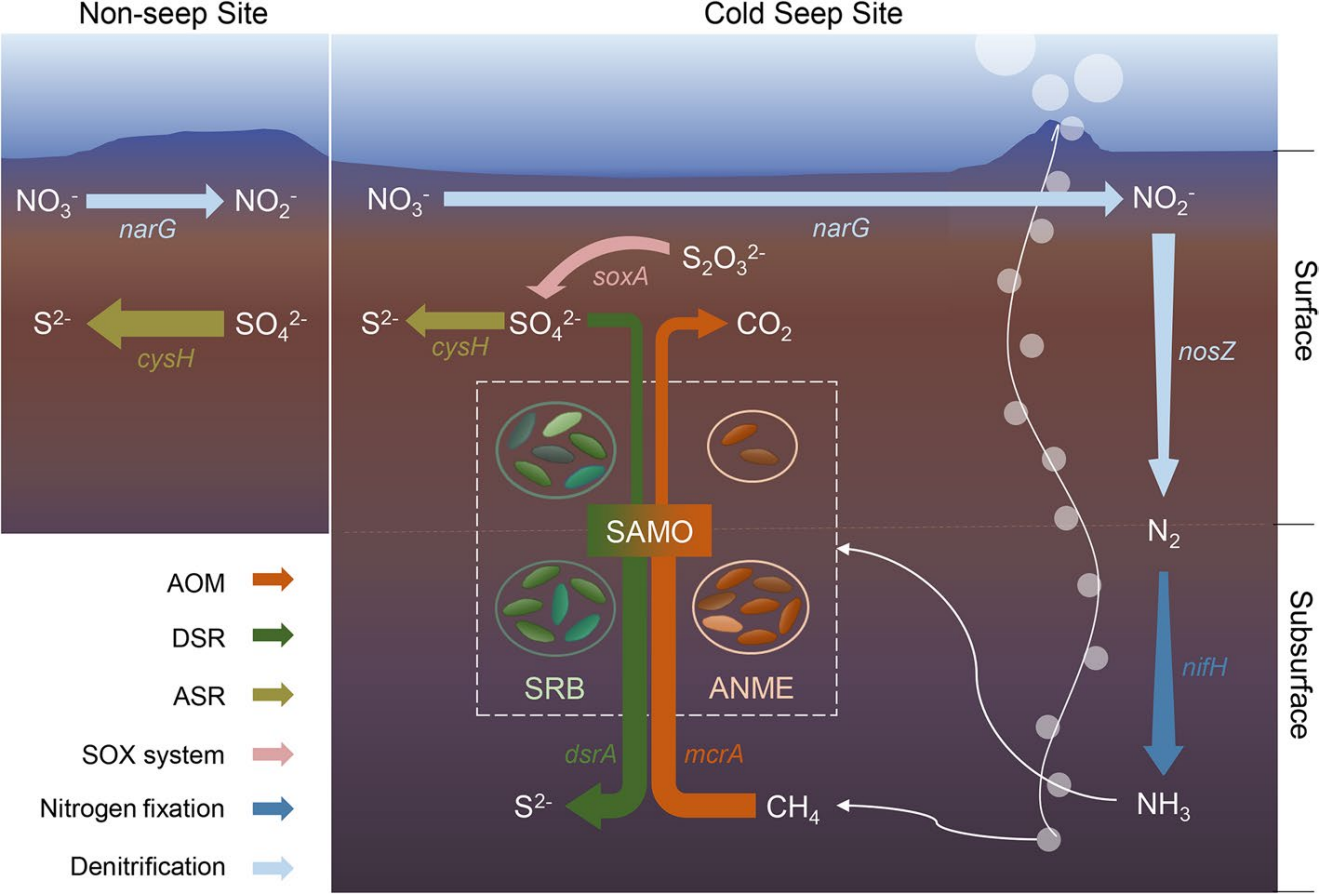
Conceptual diagram of primary metabolic processes in the cold seep and the non-seep site. The colors of the arrows represent the metabolic processes. Orange: anaerobic oxidation of methane (AOM). Green: dissimilatory sulfate reduction (DSR). Yellow-green: assimilatory sulfate reduction (ASR). Pink: sulfur-oxidizing X protein system (SOX system). Blue: nitrogen fixation. Light blue: denitrification. The investigated cold seep site exhibits typical sulfate-dependent anaerobic methane oxidation (SAMO), predominantly driven by anaerobic methanotrophic archaea (ANME) and sulfate-reducing bacteria (SRB). In the surface layer, SRB exhibits high diversity and evenness, whereas ANME are scarce. In the subsurface layer, the abundance and diversity of ANME increase, leading to the emergence of dominant SRB which can form symbiotic associations with ANME

In order to demonstrate the divergences of microbial functional profiles, we focused on six key processes of the methane cycle and the related sulfur and nitrogen cycles in the cold seep (CS) and non-seep (NS) sites, including anaerobic oxidation of methane, dissimilatory sulfate reduction, assimilatory sulfate reduction, SOX system, nitrogen fixation, and denitrification. Taking abundance, diversity, and phylogenetic diversity into consideration, we constructed a conceptual diagram of primary metabolic processes in the CS and the NS sites (Fig. 7).

Anaerobic oxidation of methane occurred almost exclusively in the CS site, especially the subsurface layers, as the methane concentration increased rapidly with depth and the abundance and diversity of *mcrA* GSCs were higher in CS2-4 than in CS1 (Figure S1a and Fig. 5b). According to the annotation results based on SILVA 138 (Data S4), ANME-1a, 1b, ANME-2a/2b, 2c, and ANME-3 groups were detected in our study, among which ANME-1 dominated and was most abundant in CS2, ANME-2a/2b was distributed evenly in subsurface layers CS2-4, and the abundance of ANME-2c increased with depth and reached a maximum in CS4, suggesting distinctive niches and geographic distribution for different ANME in the CS site.

In dissimilatory sulfate reduction (DSR), sulfate serves as an electron acceptor, while in assimilatory sulfate reduction (ASR), sulfate is used to synthesize sulfur-containing amino acids [32]. Our results revealed that the CS site possessed both pathways, while the NS site was dominated by ASR (Fig. 7). SEEP-SRB1, which was found to form a symbiotic partnership with ANME-1, exhibited the highest level of prevalence, especially in subsurface layers of the cold seep. Conversely, other free-living SRBs were present and more diverse in the surface layer. The SOX system, which is regarded as the central sulfur oxidization pathway of phototrophic and chemotrophic sulfur-oxidizing bacteria [33], was only detected in the surface layer of this cold seep.

Marine diazotrophs supply nearly one-half of the global fixed nitrogen demand, meanwhile, at least half of the ocean’s fixed nitrogen is lost by sedimentary denitrification [34, 35]. We found that the *nifH* gene related to nitrogen fixation occurred mainly in the subsurface layers of the CS site, while genes related to denitrification occurred mainly in the surface layers. More specifically, although the *narG* gene was diverse in the surface layers of both the CS and the NS site (Fig. 5e), *nosZ* was more abundant and diverse in CS1 (Figure S4k), suggesting that the denitrification occurred to a greater extent in the cold seep. Furthermore, we noted that ammonium concentration increased with depth (Figure S1e), paralleling the trends of *nifH* gene abundance. Nitrate and nitrite concentrations decreased with depth (Figure S7c and d), aligning with the trends of *narG* and *nosZ* gene abundance, and supporting the notion that diazotrophs occur in the subsurface layers while denitrification primarily occurs in the surface layer of the cold seep.

## Discussion

Bioinformatics tools for shotgun metagenome datasets are continually improving, and the most commonly used methods can be divided into read-based and assembly-based systems. Read-based methods, which directly map the original reads to reference databases, have the potential to perform large-scale analyses due to their simplicity. Recently, several emerging databases, such as MCycDB, SCycDB, and NCycDB, are expected to improve the searching efficiency and accuracy of read-based analysis [23–25]; however, this still relies on the completeness and accuracy of the known reference genes. On the other hand, assembly-based methods firstly assemble reads into contigs. Then, after taxonomic and functional annotation, reads are mapped to these annotated contigs. For instance, assembly-based methods have been used to investigate the whole microbial community and their ecological functions in deep-sea cold seep ecosystems [14]. It is worth emphasizing that phylogenetic diversity is a useful measure to consider taxonomic changes that are linked to niche differentiation, resource partitioning, and other ecological processes. However, read-based methods are usually unable to investigate phylogenetic relationships, as they only map reads to an existing database, and are unable to obtain full-length genes. Additionally, for assembly-based methods, sufficient gene coverage is difficult to obtain, especially for environmental functional groups [36], and thus the phylogenetic tree of the retrieved functional genes could be incomplete. Our method is a combination of read-based and assembly-based methods. With protein prediction based on assembled contigs and iteration based on reads, our method fully mined the potential information of metagenomics data, identified subtle variations in phylogenetic diversity that conventional methods are unable to discern, and expanded our knowledge of functional groups in this extreme environment.

According to the level of specificity at which functional diversity is analyzed, methods also can be classified into targeted and non-targeted [37]. Targeted methods are typically used to measure the diversity of a single specific functional trait, such as estimating the diversity of sequence variants of a specific gene or protein. This type of method is highly specific and has been used to recover *mcrA* gene diversity and analyze the *nifH* gene in-depth [16, 38]. Although it is appropriate for focused subjects and hypotheses, it might be difficult to observe potential relationships between taxa. Non-targeted approaches, on the other hand, capture a wide breadth of functional traits and have been used to reveal nitrogen, methane, and sulfur metabolism in Yellowstone hot spring samples [39]. This type of method focuses on metabolic pathways instead of the diversity of individual genes, resulting in a general functional profile but may ignore critical details. Overall, targeted and non-targeted methods are two extremes of specificity and breadth. Our approach considers both the breadth and depth of functional genes, through which we analyzed the abundance profiles of 68 representative functional genes at a non-seep site and five cold seep sites, and then explored the phylogeny of specific genes. It provided insights into the most representative functional genes and details about the functional groups in cold seeps.

Previous studies on cold seeps were mainly concerned with the distribution of microbial communities over large vertical scales, as the biological removal of methane by SAMO always occurs at a distinct deep zone known as the sulfate-methane transition zone [11, 13, 40]. However, the extreme spatial heterogeneity at the scale of a few centimeters in cold seeps was also confirmed [41]. The microbial activity in shallow sediment has a more direct impact on global element cycling, making it crucial for comprehending the functioning of cold seep ecosystems. We investigated the cold seep sediments at a smaller but finer vertical scale, uncovering a shallow SMTZ. Our findings revealed that even at small scales, microbial communities exhibited distinct niche separation and metabolic preferences within this zone. The subsurface layers were dominated by the SAMO process, while the surface layer of the CS site had all six key pathways, and the diversity and evenness of the SSU rRNA gene were high (Fig. 5a). The mean nearest taxon distance (MNTD) result also revealed that the MNTD value of CS1 was the highest, indicating the taxa in the surface layer were more phylogenetically distant (Figure S7). In the surface layer, gas and solute exchanges at the water–sediment interface are generally active due to the large gas fluxes and bioturbation, which have impacts on the microbial communities [41]. Considering the complex and dynamic environment, communities in the surface layer developed high species richness and evenness, as well as diverse metabolic pathways and strong resistance, to maintain community stability. Therefore, the surface layer of the CS site may act as a reservoir of microbial species and functions.

Microbial communities exhibited pronounced dissimilarity both among non-seep and cold seep sites, as well as across various cold seep locations. The conceptual diagram in Fig. 7 portrays the specific scenario of the CS site and the adjacent NS site, and may not be universally applicable to all cold seeps. Notably, even HM1 and HM5, situated in the Haima cold seep and sharing a similar geographic location with CS, do not align with the depicted conceptual diagram. HM1 had a higher abundance and diversity of functional genes associated with sulfate and nitrate metabolism, whereas nitrate reduction genes were absent in HM5. Interestingly, despite variations in the richness and abundance of the *mcrA* gene, its phylogenetic diversity demonstrated comparability across these three sites, implying that while ANME had experienced distinct evolution and selection, microbial communities in the Haima cold seep preserved similar potentials for AOM. Local diversification in cold seep sites could be affected by methane-supply regimes, gradients of electron acceptors and donors, and biological interactions [42]. Thus, environmental factors, both abiotic and biotic, had a complex influence on functional groups. In-depth interpretation is required in combination with more environmental factor data and analytical methods. In addition, we found that coupling occurs between specific GSCs, but we are uncertain whether this phenomenon is derived from the fact that functional groups with specific GSCs are more likely to establish symbiotic relationships, or that long-term symbiotic relationships select specific GSCs. To address this, time series studies, synthetic community experiments, and other analyses are required.

## Conclusions

Our pipeline integrates both read-based and assembly-based techniques while maintaining a balance between targeted and non-targeted methods. It has proven to be robust to errors and omissions in the reference database. By applying REMIRGE to the cold seep dataset, we have been able to gain deeper insights into the phylogeny and achieve a more comprehensive assessment of microbial diversity. These findings have opened exciting avenues for further research. To enhance user-friendliness, necessary scripts and a convenient online analysis platform are made available. With the potential to explore a wide range of environments, this innovative pipeline holds great promise in advancing our understanding of functional groups and their contributions to ecosystem processes.

## Materials and methods

### Experimental design

In 2019, sediment samples were collected from the Haima cold seep (− 1371 m) using a push-core. As shown in Fig. 2a, the sediment core was collected from a site of active methane seepage, and equally divided into four sections (groups), as follows: CS1 (0–6 cm), CS2 (6–12 cm), CS3 (12–18 cm), and CS4 (18–24 cm). Meanwhile, the core of the control group was collected from an adjacent non-seep site, about 6.73 m away from the seep site, and equally divided into four sections as well. Then each layer was equally divided into five parts. All 40 samples were delivered to the laboratory on dry ice and stored at − 80 °C to decrease microbial activity. After 24 h of vacuum freeze-drying, microbial community genomic DNA was extracted from 2 g of freeze-dried sediment with the grind plus kit method as described previously [43]. The DNA was quantified with Qbuit® 3.0, and the quality was checked via gel electrophoresis. As the DNA concentrations of the non-seep site’s subsurface layers were too low for sequencing, only the surface layer of the non-seep site (NS, 0–6 cm) was retained as the control group. Both 2nd generation (NovaSeq) and 3rd generation (Nanopore) technologies were applied to perform metagenomic sequencing at private companies (Guangdong Magigene Biotechnology Co. Ltd. and Wuhan Benagen Technology Co., Ltd., respectively). For NovaSeq, sequencing libraries were generated using NEB Next® Ultra™ DNA Library Prep Kit for Illumina® following the manufacturer’s recommendations, and index codes were added. The library quality was assessed on the Qubit® 4.0 Fluorometer and Qsep400 High-Throughput Nucleic Acid Protein Analysis system. Finally, the library was sequenced on an Illumina NovaSeq 6000 platform and 250 bp paired-end reads were generated. Meanwhile, five DNA replicates of each group were equally mixed and then purified using Agencourt Ampure XP beads. Next, a prepared library was loaded on the PromethION Flow Cell R9.4 and transferred into Oxford Nanopore PromethION for sequencing.

Another 24 cm sediment core was divided into 12 layers to measure environmental factors. Concentrations of methane, sulfate, nitrate, nitrite, ammonium, and dissolved inorganic carbon (DIC), as well as the δ^13^C value of DIC, were determined using previously established methods [21].

### Quality control and assembly

FastUniq (version 1.1) [44] was used to remove PCR duplications, and Trimmomatic (version 0.36) [45] was used to trim and filter raw short reads (parameters: -threads 16 -phred33 LEADING:3 TRAILING:3 SLIDINGWINDOW:4:20 MINLEN:50). Next, the quality of clean reads was evaluated by FastQC (version 0.11.8). For each group, the five replicate FASTQ files were merged for subsequent assembly. NS, CS1, CS2, and CS3 were assembled using metaSPAdes (version 3.14.1) [46] with default parameters, while CS4 was assembled using Megahit (version 1.2.9) [47] due to the larger number of reads. Next, long reads generated by Nanopore sequencing and OPERA-MS (version 0.9.0) [48] were used for hybrid metagenomics assembly to improve the contiguity of contigs.

In addition, four other metagenomic reads datasets (HY, SF, HM1, and HM5) were downloaded from NCBI, please see Data S1 for more details. The quality control methods were the same as above.

### Fast profiling of functional genes

MCycDB, SCycDB, and NCycDB, which are the specialized databases for the representative functional genes in methane, sulfur, and nitrogen cycling, were used for fast profiling of functional genes in the non-seep and five cold seep sites [23–25]. We selected 68 representative functional genes, including 23 for the methane cycle, 23 for the sulfur cycle, and 22 for the nitrogen cycle, to study the functional profiles. We calculated the total abundance of each gene and the respective abundances of the samples. After natural log transformation, R and RStudio were used to create the heatmap with the pheatmap package (version 1.0.12). For NS and CS1-4, the abundance of functional genes in the five replicates of each layer was also calculated. The significance was evaluated using the Kruskal–Wallis test and Dunn's all-pairs rank comparison test (PMCMRplus package, version 1.9.6), and the *P* values were corrected by the false discovery rate (FDR) method.

### REMIRGE improvements and applications

We modified the EMIRGE script [6] for building the starting reference database based on functional genes. After that, we could input our reference database of functional genes of interest (FASTA format), and the script would filter and cluster sequences, deal with ambiguous bases, and finally create a starting bowtie2 index. Furthermore, we also modified the EMIRGE script for iteration, and replaced the older version of Bowtie with the updated version, Bowtie2 [49]. Therefore, some parameters such as insert size distribution mean and distribution standard deviation were no longer necessary. During iteration, if the fraction of variants in a GSC exceeded 0.04, a new GSC was split out; if the identity of two GSCs exceeded 0.97, they were merged into one.

Prokka (version 1.14.6) [50] was used to predict and annotate functional genes from the assembled contigs with two modes (-Archaea and -Bacteria) and the default databases. After that, the REMIRGE was applied for analyzing the SSU rRNA gene, and functional genes. The initial database of the SSU rRNA gene was SILVA 138 SSU Ref NR99 [51], and the initial database for functional genes was from Prokka annotation (*mcrA* and *dsrA* were also derived from FunGene) [19]. Simultaneously, clean reads were tagged, merged, and input for iterations with default parameters (-n 40 -a 1 -p 0.04 -v 0.1 -j 0.97 -c 3 –phred33). After iterations, a final bam file was generated, recording which read matched with which reference sequence. The workflow is shown in Figure S8. We wrote python scripts for abundance calculation, and the abundance was normalized by the equation below:

$${N}_{s,i}={A}_{s,i}\times \frac{{r}_{\mathrm{max}}}{{r}_{s}}\times \frac{{l}_{\mathrm{max}}}{{l}_{i}}$$

where ${A}_{s,i}$ stands for the original abundance, ${N}_{s,i}$ stands for the normalized abundance of gene *i* in sample *s*, ${r}_{s}$ stands for the reads number of sample*s*, ${r}_{max}$ stands for the highest reads number among all samples, ${l}_{i}$ stands for the length of gene *i*, and ${l}_{\mathrm{max}}$ stands for the length of the longest gene.

Considering that CS4_3 varied greatly from the other replicates of CS4, it was removed from subsequent analyses. A well-organized *dsrAB* database was used to classify *dsrA* genes with BLAST (version 2.11.0 +) [52], including their types (oxidative or reductive), environmental distributions, and taxa [29].

By inputting clean reads of the CS site to REMIRGE, we obtained the real full-length sequences of functional genes at the site after iteration. Subsequently, these sequences were input as the starting reference database to reconstruct the real functional gene sequences from the other cold seep sites (HM1, HM5, HY, and SF). The abundance was calculated in the same manner as above.

### Phylogenetic tree construction

MAFFT (version 7.310) [53] was used to align the SSU rRNA genes. Whereas for functional genes, MACSE (version 2.05) [54] was used to align the coding sequences based on their amino acid translations while accounting for frameshifts. After that, FastTree (version 2.1.10) [55] was used to infer phylogenies for previously aligned sequences. The tree files were uploaded to Interactive Tree Of Life (https://itol.embl.de) for visualization [56]. In addition, we used an online analysis platform (https://dmap.denglab.org.cn) [57] to annotate SSU rRNA gene sequence clusters [58], and calculated the abundance of several critical phyla. We also used the platform to calculate the mean nearest taxon distance (MNTD) of the SSU rRNA GSCs in each sample. Significant differences between groups were evaluated using Dunn’s test, and the *P* values were corrected by the FDR method via the FSA package (version 0.9.3).

### Diversity and correlation analysis

Alpha diversity indexes of the SSU rRNA gene and each functional gene were evaluated by Hill numbers with gradient orders (*q* = 0, 1, 2), including diversity ^*q*^*D* and phylogenetic diversity ^*q*^*PD* [26]. Abundance matrices were used for ^*q*^*D* calculation, while abundance matrices and tree files were used for ^*q*^*PD* calculation. Calculations were conducted in R and RStudio, with the hilldiv package (version 1.5.1) [59] for ^*q*^*D* while the hillR package (version 0.5.1) [60] for ^*q*^*PD*. For beta diversity, we employed the phyloseq (version 1.38.0) to calculate the weighted-UniFrac distance [61, 62], and ggplot2 (version 3.3.6) [63] for principal coordinates analysis (PCoA) plots. PERMANOVA based on weighted UniFrac distance dissimilarities was conducted using the vegan package (version 2.6–2) and the pairwiseAdonis package (version 0.4), and the *P* values were corrected by FDR. Moreover, the correlation was performed using Pearson correlation analysis. Correlations between functional genes were visualized by the corrplot package (version 0.92), while correlations between environmental factors were visualized by ggplot2 (version 3.3.6). Mantel analysis between GSCs and environmental factors was conducted using the function mantel of the vegan package (version 2.6–2). For GSCs, we calculated the weighted Unifrac distance between samples in conjunction with the phylogenetic tree; for environmental factors, we calculated the Euclidean distance between samples.

### Online analysis platform of REMIGE

We developed an online analysis platform for REMIRGE (https://remirge.denglab.org.cn). The “REMIRGE” section includes the main program of REMIRGE, as well as tools for abundance statistics and normalization, which are designed for processing output files of REMIRGE. The “Phylogenetic diversity” section integrates alignment tools including MAFFT and MACSE, FastTree for inferring phylogenies, and scripts for calculating diversity and phylogenetic diversity profiles. The “Miscellaneous” section contains some file processing tools. Please refer to the user manual on the website for more guidance.

### Supplementary Information

**Additional file 1: Figure S1.** Regression analysis of environmental factors in Haima cold seep sediments. a Methane (μM), b Sulfate (mM), c Nitrate(μM), d Nitrite(μM), e Ammonium(μM), f DIC (mM) and g δ^13^C_DIC_ (‰). **Figure S2.** Principal coordinates analysis (PCoA) plots of representative genes based on weighted-Unifrac distance. The shapes and colors of the data points correspond to various cold seep sites, while the size reflects the average sampling depth. Ellipses represent 95% confidence intervals. a *mcrA*, b *dsrA*, c *nifH*, d *narG*, and e *nosZ*. Overall *P*-values are annotated on the plots, while pairwise *P*- and F- values are compiled in Data. S3. **Figure S3.** Principal coordinates analysis (PCoA) plots of other significant genes based on weighted-Unifrac distance. Ellipses represent 95% confidence intervals. a *mtrA*, b *mer*, c *mtd*, d *mch*, e *ftr* and f *fwdC* for methane cycle; g *sat*, h *aprA*, i *sir*, and j *soxA* for sulfur cycle; k *nosZ* for nitrogen cycle. Overall *P*-values are annotated on the plots, while pairwise *P*- and F- values are compiled in Data. S3. **Figure S4.** Phylogenetic trees, abundance heat maps and diversity profiles of other significant functional genes. Only branches whose supporting values were higher than 0.75 are represented in the phylogenetic trees. The outer heat maps illustrate logarithmically transformed absolute abundance. The inner strips represent the types of gene sequence clusters (GSCs), while the heat maps at bottom-right inset for each gene show the diversity profiles measured by Hill numbers, logarithmically transformed and scaled by row. a *mtrA*, b *mer*, c *mtd*, d *mch*, e *ftr* and f *fwdC* for methane cycle; g *sat*, h *aprA*, i *sir*, and j *soxA* for sulfur cycle; k *nosZ* for nitrogen cycle. **Figure S5.** Pearson correlation analysis between *narG* and *mcrA*. **Figure S6.** Pearson correlation analysis between a *dsrA* and *nifH*; b *mcrA* and *nifH*. **Figure S7.** Box plots of mean nearest taxon distance (MNTD) of SSU rRNA gene sequence clusters, significance tests are at 5% significance level. The letters above the boxes show the significance, and different letters indicate significant differences. **Figure S8.** Metagenomic data analysis workflow. **Table S1.** Mantel test between environmental factors and functional genes (Pearson correlation coefficient based on weighted Unifrac distance).

**Additional file 2: Data S1.** Sample information.

**Additional file 3: Data S2.** Dunn's non-parametric pairwise comparison test for Kruskal-type ranked data. The *P*-values were corrected by FDR method. Significant values (5% significance level) are colored light red.

**Additional file 4: Data S3.** Pairwise PERMANOVA results based on weighted-UniFrac distance dissimilarities. The values of upper triangular matrices are *P*-values, while the values of lower triangular matrices are F-values. The *P*-values were corrected by FDR method. Significant values (5% significance level) are colored light red.

**Additional file 5: Data S4.** Taxonomic annotations of SSU rRNA gene sequence clusters according to SILVA 138 database.

## Data Availability

The metagenomics data reported in this paper have been deposited in the Genome Sequence Archive in the National Genomics Data Center, China National Center for Bioinformation / Beijing Institute of Genomics, Chinese Academy of Sciences that are publicly accessible at https://ngdc.cncb.ac.cn/gsa (GSA: CRA009925 for NovaSeq and CRA009932 for Nanopore). All scripts for the reprogrammed EMIRGE, abundance calculations, and normalization are available on GitHub (https://github.com/yedeng-lab/Reprogrammed-EMIRGE).
