# Supplementary material for: Phylogenetic diversity of functional genes in deep-sea cold seeps: a novel perspective on metagenomics
Source: Microbiome. 2023 Dec 15;11:276. doi: 10.1186/s40168-023-01723-7 (PMC10722806; doi:10.1186/s40168-023-01723-7)
Supplement: Supplementary file 2 — Additional file 1: Figure S1. Regression analysis of environmental factors in Haima cold seep sediments. a Methane (μM), b Sulfate (mM), c Nitrate(μM), d Nitrite(μM), e Ammonium(μM), f DIC (mM) and g δ13CDIC (‰). Figure S2. Principal coordinates analysis (PCoA) plots of representative genes based on weighted-Unifrac distance. The shapes and colors of the data points correspond to various cold seep sites, while the size reflects the average sampling depth. Ellipses represent 95% confidence intervals. a mcrA, b dsrA, c nifH, d narG, and e nosZ. Overall P-values are annotated on the plots, while pairwise P- and F- values are compiled in Data. S3. Figure S3. Principal coordinates analysis (PCoA) plots of other significant genes based on weighted-Unifrac distance. Ellipses represent 95% confidence intervals. a mtrA, b mer, c mtd, d mch, e ftr and f fwdC for methane cycle; g sat, h aprA, i sir, and j soxA for sulfur cycle; k nosZ for nitrogen cycle. Overall P-values are annotated on the plots, while pairwise P- and F- values are compiled in Data. S3. Figure S4. Phylogenetic trees, abundance heat maps and diversity profiles of other significant functional genes. Only branches whose supporting values were higher than 0.75 are represented in the phylogenetic trees. The outer heat maps illustrate logarithmically transformed absolute abundance. The inner strips represent the types of gene sequence clusters (GSCs), while the heat maps at bottom-right inset for each gene show the diversity profiles measured by Hill numbers, logarithmically transformed and scaled by row. a mtrA, b mer, c mtd, d mch, e ftr and f fwdC for methane cycle; g sat, h aprA, i sir, and j soxA for sulfur cycle; k nosZ for nitrogen cycle. Figure S5. Pearson correlation analysis between narG and mcrA. Figure S6. Pearson correlation analysis between a dsrA and nifH; b mcrA and nifH. Figure S7. Box plots of mean nearest taxon distance (MNTD) of SSU rRNA gene sequence clusters, significance tests are at 5% sign [file 40168_2023_1723_MOESM1_ESM.docx]

**Supporting Information for**

Phylogenetic diversity of functional genes in deep-sea cold seeps: a novel perspective on metagenomics

Danrui Wang^1, 2^, Jiangtao Li^3^, Lei Su^3^, Wenli Shen^1, 4^, Kai Feng^1, 2^, Xi Peng^1, 2^, Zhujun Wang^5^, Bo Zhao^1, 2^, Zheng Zhang^4^, Zhaojing Zhang^4^, Étienne Yergeau^6^, Ye Deng^1, 2, 4^

* Ye Deng

Email: [yedeng@rcees.ac.cn](mailto:yedeng@rcees.ac.cn)

**This PDF file includes:**

Figure S1 to S8

Table S1

**Other supporting materials for this manuscript include the following:**

Data S1 to S4


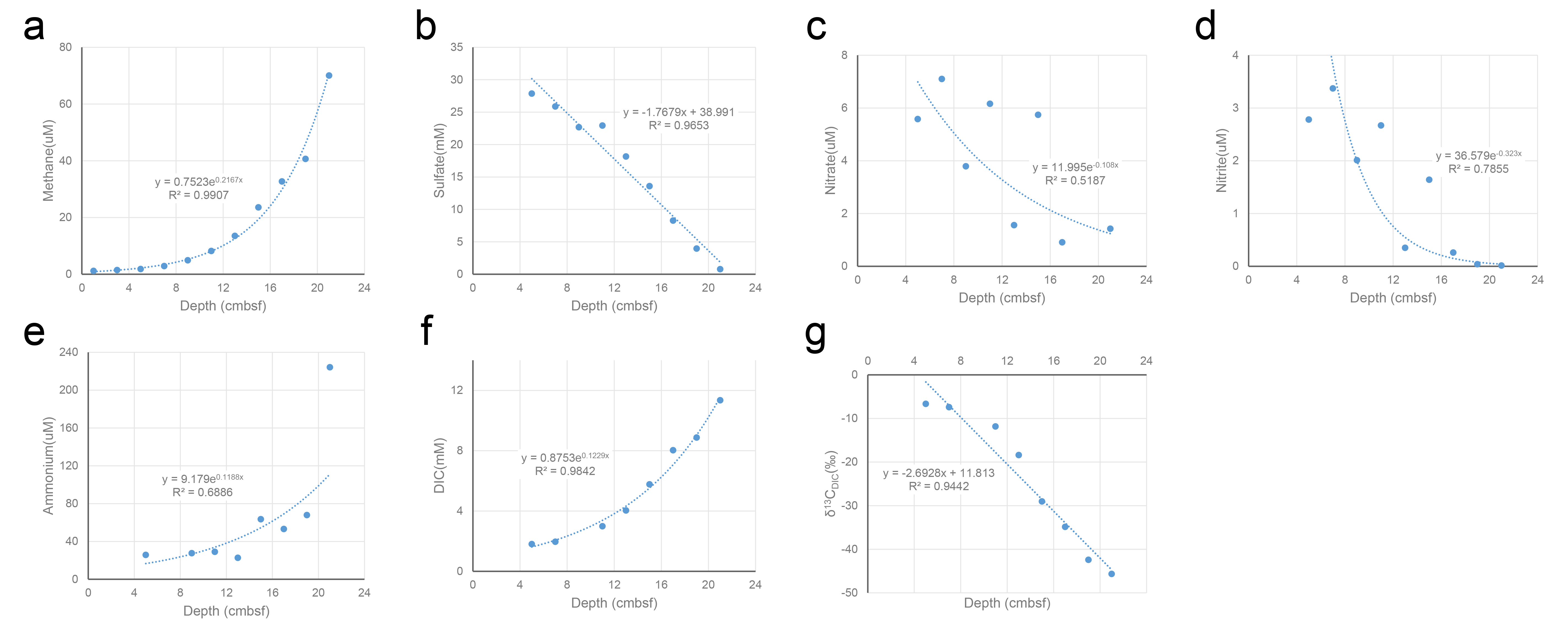


**Figure S1. Regression analysis of environmental factors in Haima cold seep sediments. a** Methane (μM)**, b** Sulfate (mM), **c** Nitrate(μM), **d** Nitrite(μM), **e** Ammonium(μM), **f** DIC (mM) and **g** δ^13^C_DIC_ (‰).


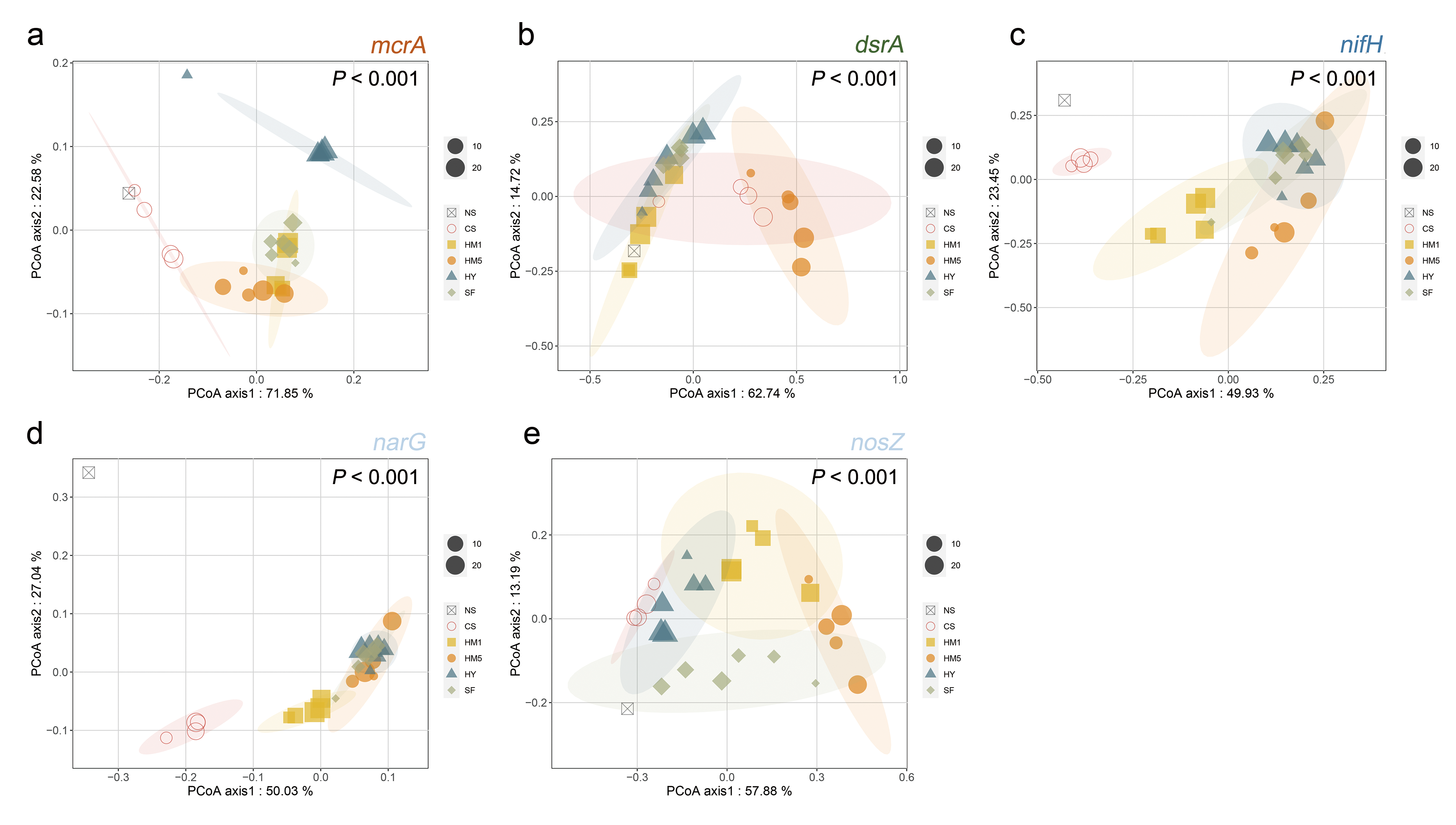


Figure S2. Principal coordinates analysis (PCoA) plots of representative genes based on weighted-Unifrac distance. The shapes and colors of the data points correspond to various cold seep sites, while the size reflects the average sampling depth. Ellipses represent 95% confidence intervals. a *mcrA*, b *dsrA*, c *nifH*, d *narG*, and e *nosZ*. Overall *P*-values are annotated on the plots, while pairwise *P*- and F- values are compiled in Data. S3.


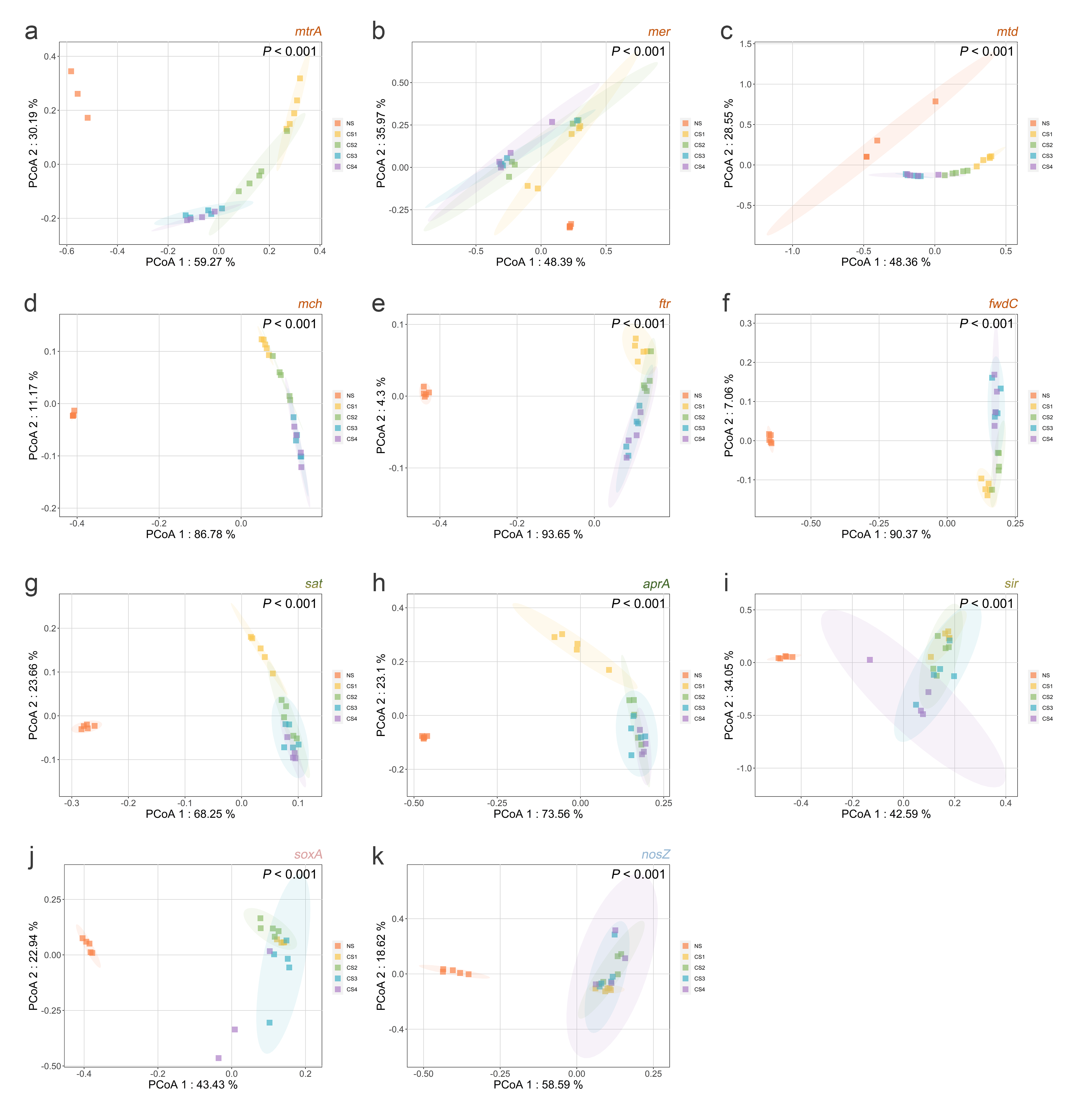


Figure S3. Principal coordinates analysis (PCoA) plots of other significant genes based on weighted-Unifrac distance. Ellipses represent 95% confidence intervals. a *mtrA*, b *mer*, c *mtd*, d *mch*, e *ftr* and f *fwdC* for methane cycle; g *sat*, h *aprA*, i *sir*, and j *soxA* for sulfur cycle; k *nosZ* for nitrogen cycle. Overall *P*-values are annotated on the plots, while pairwise *P*- and F- values are compiled in Data. S3.


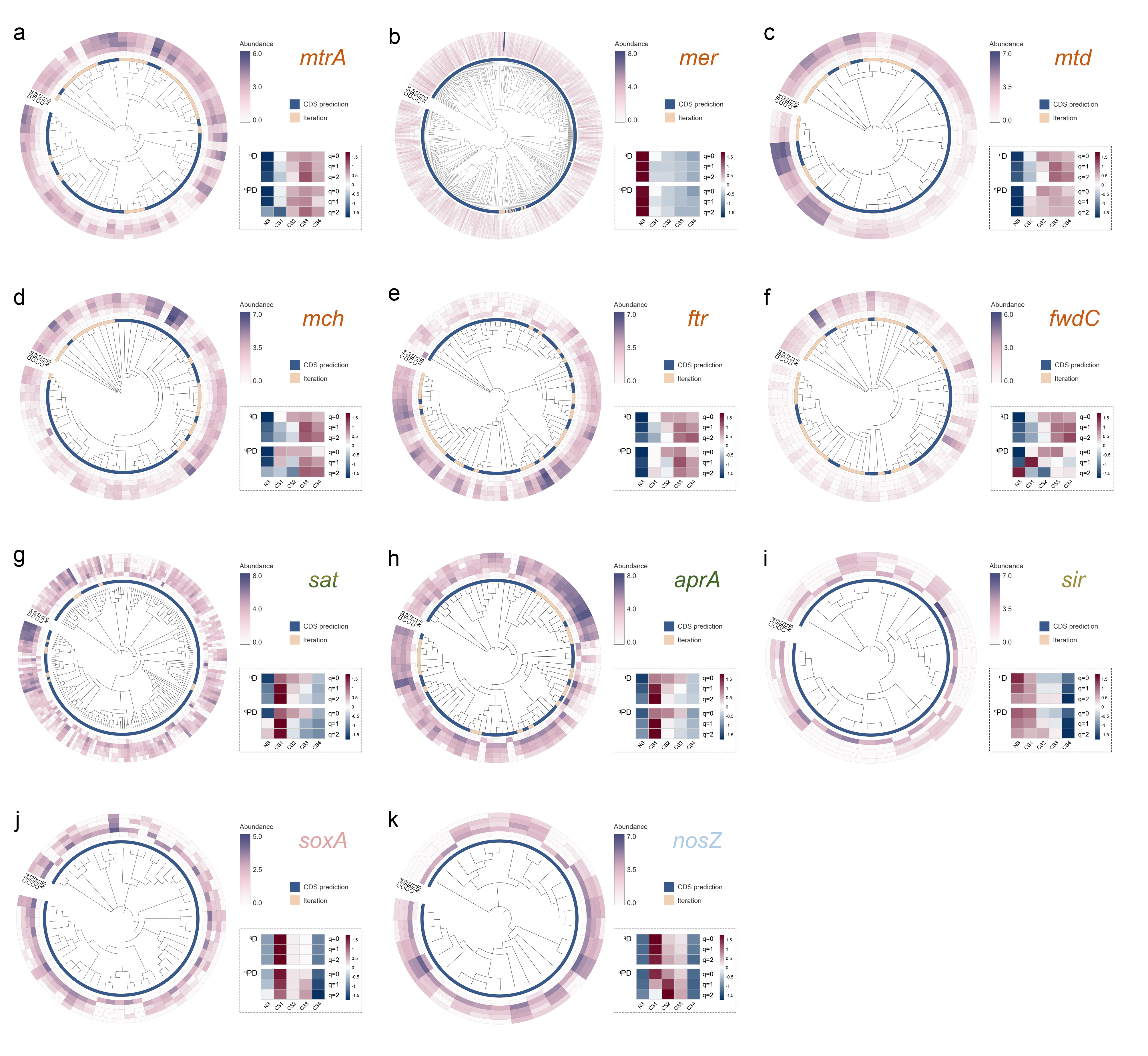


Figure S4. Phylogenetic trees, abundance heat maps and diversity profiles of other significant functional genes. Only branches whose supporting values were higher than 0.75 are represented in the phylogenetic trees. The outer heat maps illustrate logarithmically transformed absolute abundance. The inner strips represent the types of gene sequence clusters (GSCs), while the heat maps at bottom-right inset for each gene show the diversity profiles measured by Hill numbers, logarithmically transformed and scaled by row. a *mtrA*, b *mer*, c *mtd*, d *mch*, e *ftr* and f *fwdC* for methane cycle; g *sat*, h *aprA*, i *sir*, and j *soxA* for sulfur cycle; k *nosZ* for nitrogen cycle.


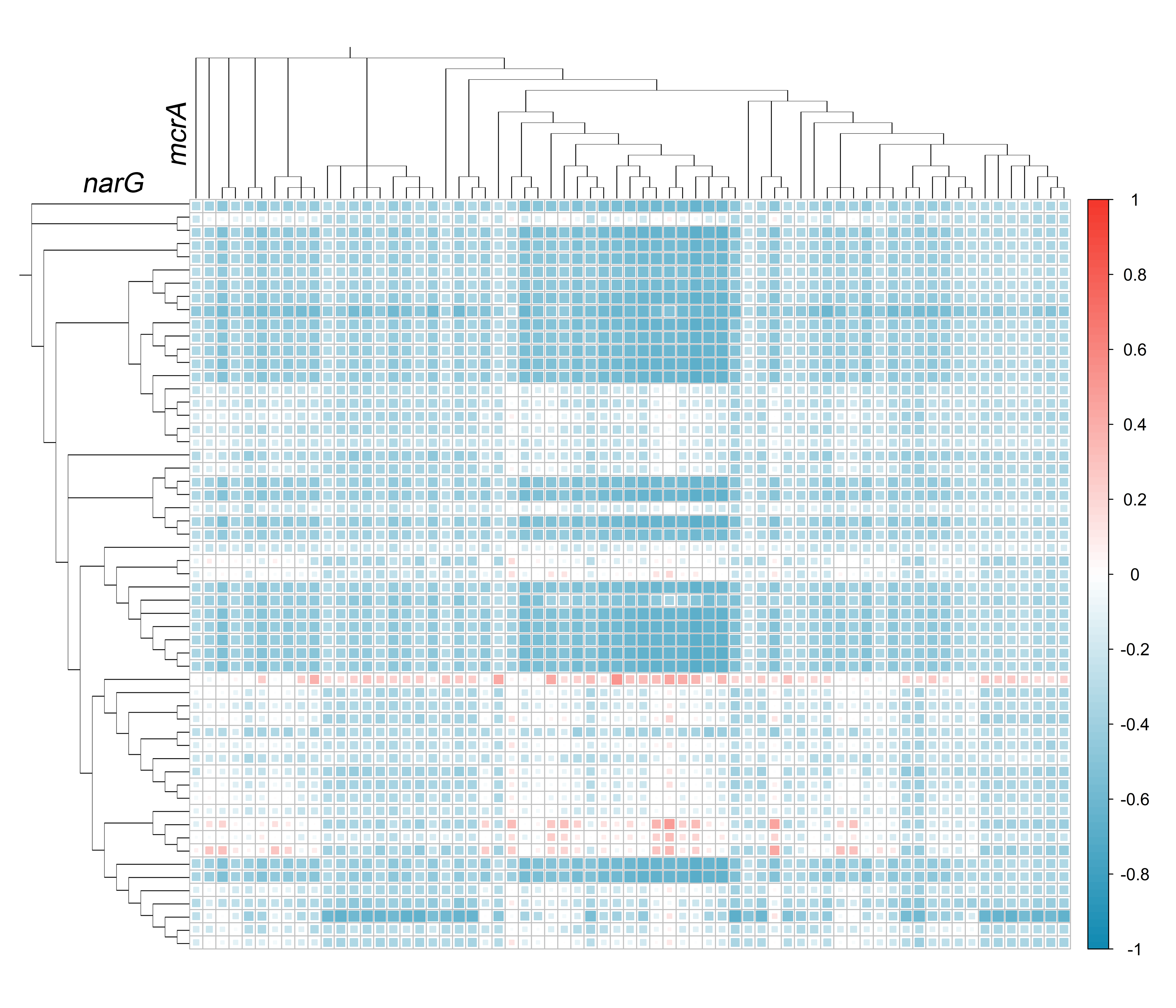


Figure S5. Pearson correlation analysis between *narG* and *mcrA*.





Figure S6. Pearson correlation analysis between a *dsrA* and *nifH*; b *mcrA* and *nifH*.


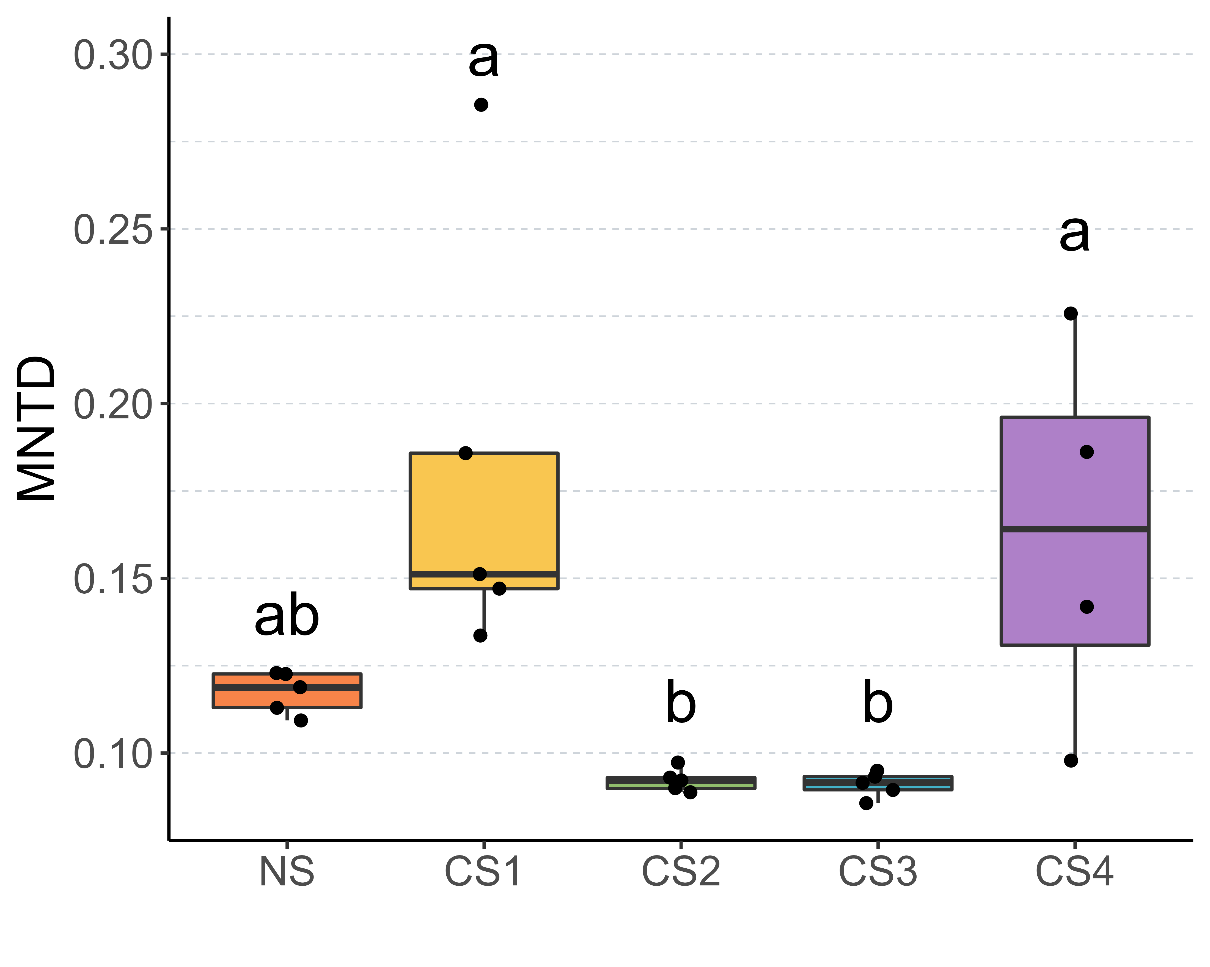


Figure S7. Box plots of mean nearest taxon distance (MNTD) of SSU rRNA gene sequence clusters, significance tests are at 5% significance level. The letters above the boxes show the significance, and different letters indicate significant differences.


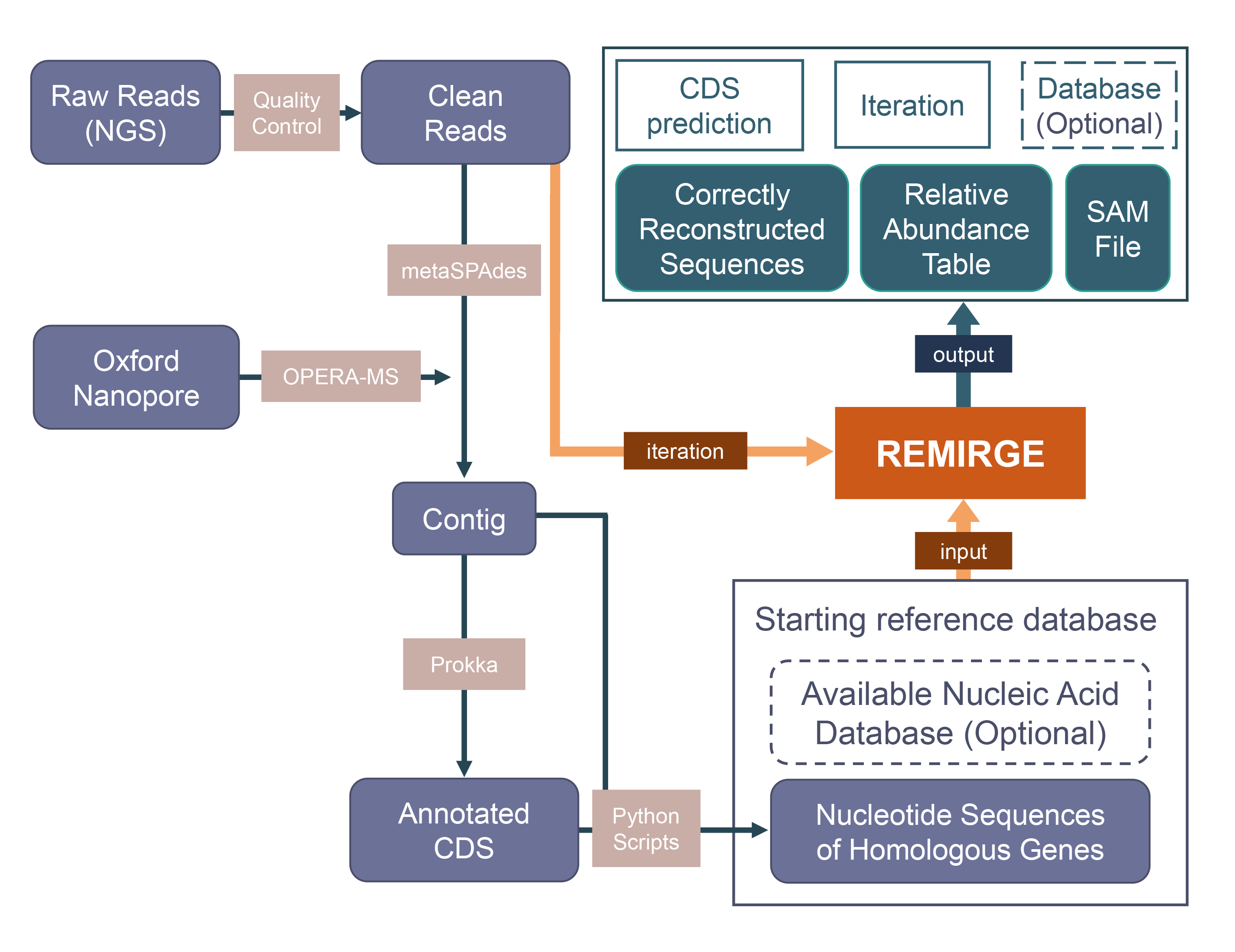


Figure S8. Metagenomic data analysis workflow.

**Table S1** Mantel test between environmental factors and functional genes (Pearson correlation coefficient based on weighted Unifrac distance).

| Environmental Factor | *mcrA* | | *dsrA* | | *soxA* | | *cysH* | | *nifH* | | *narG* | | *nosZ* | |
| --- | --- | --- | --- | --- | --- | --- | --- | --- | --- | --- | --- | --- | --- | --- |
|  | r | *p* | r | *p* | r | *p* | r | *p* | r | *p* | r | *p* | r | *p* |
| Methane | **0.390855** | **0.003** | 0.139298 | 0.111 | **0.552319** | **0.002** | **0.485635** | **0.001** | 0.074854 | 0.222 | **0.594062** | **0.001** | **0.416983** | **0.009** |
| Sulfate | **0.727673** | **0.001** | **0.593486** | **0.001** | **0.378269** | **0.001** | **0.707469** | **0.001** | **0.503632** | **0.001** | **0.498841** | **0.001** | **0.322083** | **0.005** |
| Ammonium | **0.301826** | **0.007** | 0.116099 | 0.141 | **0.543532** | **0.002** | **0.435173** | **0.001** | 0.04021 | 0.297 | **0.598466** | **0.004** | **0.436947** | **0.009** |
| Nitrate | **0.445432** | **0.001** | **0.63583** | **0.001** | **0.239731** | **0.019** | **0.505553** | **0.001** | **0.532842** | **0.001** | **0.367311** | **0.001** | **0.27686** | **0.005** |
| Nitrite | **0.648523** | **0.001** | **0.897129** | **0.001** | -0.06043 | 0.646 | **0.56524** | **0.001** | **0.787763** | **0.001** | 0.13618 | 0.133 | 0.080521 | 0.236 |
| X13DIC | **0.776451** | **0.001** | **0.603294** | **0.001** | **0.36272** | **0.003** | **0.724059** | **0.001** | **0.524716** | **0.001** | **0.471298** | **0.001** | **0.290304** | **0.006** |
| DIC | **0.53687** | **0.002** | **0.286179** | **0.028** | **0.519436** | **0.004** | **0.584273** | **0.001** | **0.217337** | **0.045** | **0.579439** | **0.001** | **0.391476** | **0.002** |

**Data S1 (separate file).** Sample information.

Data S2 (separate file). Dunn's non-parametric pairwise comparison test for Kruskal-type ranked data. The *P*-values were corrected by FDR method. Significant values (5% significance level) are colored light red.

Data S3 (separate file). Pairwise PERMANOVA results based on weighted-UniFrac distance dissimilarities. The values of upper triangular matrices are *P*-values, while the values of lower triangular matrices are F-values. The *P*-values were corrected by FDR method. Significant values (5% significance level) are colored light red.

Data S4 (separate file). Taxonomic annotations of SSU rRNA gene sequence clusters according to SILVA 138 database.
